# Longitudinal relationships among early adolescent physical exercise, internalizing symptoms, and learning engagement: exploring within-person dynamics and the role of gender differences

**DOI:** 10.1186/s13034-025-00965-7

**Published:** 2025-09-29

**Authors:** Bohang Wei, Wenyuan Jiang, Jianfeng Liu, Jingtao Wu, Chunmeng Xu, Yujiang Guo, Mingjun Xie

**Affiliations:** 1 School of Physical Education (main campus), Zhengzhou University, Zhengzhou, China; 2 Department of Student Affairs, Shandong College of Traditional Chinese Medicine, Yantai, China; 3 Department of Sports Training, Henan Sport University, Zhengzhou, China; 4 School of Physical Education, Leshan Normal University, Leshan, China; 5 Jiying Middle School, Kaifeng, China; 6 Faculty of Psychology, Beijing Normal University, Beijing, China; 7 Department of Martial Arts and Ethnic Traditional Sports, Henan Sport University, Zhengzhou, China; 8 Institute of Developmental Psychology, Beijing Normal University, Beijing, China

**Keywords:** Physical exercise, Internalizing symptoms, Learning engagement, Early adolescents, Random intercept cross-lagged panel model, Gender differences

## Abstract

**Background:**

Current research lacks comprehensive investigation into the longitudinal relationships among physical exercise (PE), internalizing symptoms (IS), and learning engagement (LE) from an integrated perspective. Particularly scarce are examinations of underlying mediating mechanisms and gender differences within these longitudinal relationships.

**Aims:**

(1) To adopt a comprehensive approach in examining the within-person relationships among PE, IS, and LE, as well as their potential mediating mechanisms; (2) To investigate whether gender differences exist in the relationships among these three variables.

**Sample and methods:**

A one-year longitudinal study with 1,601 Chinese junior high school students assessed PE, IS, and LE at three time points (T1: December 2023, T2: June 2024, T3: December 2024). Random intercept cross-lagged panel models (RI-CLPM) and multi-group analyses were conducted.

**Results:**

RI-CLPM results revealed that: (1) T1 PE positively predicted T2 LE, and T2 PE positively predicted T3 LE; (2) T1 LE negatively predicted T2 IS; (3) T1 IS negatively predicted T2 LE, T2 IS negatively predicted T3 LE, and T2 LE negatively predicted T3 IS, confirming bidirectional relationships; (4) T2 IS longitudinally mediated between T1 PE and T3 LE. The results of gender-based multi-group comparison showed that: (1) autoregressive pathway of T1 PE to T2 PE was only significant in boys; (2) the autoregressive path of T2 IS to T3 IS was only significant in girls; (3) The positive prediction path of T1 PE on T2 LE was only significant in boys, and the positive prediction path of T2 PE on T3 LE was only significant in boys. (4) The negative prediction path of T2 LE on T3 IS was only significant in boys. (5) In both boys and girls, T2 IS plays a longitudinal mediating role between T1 PE and T3 LE.

**Conclusions:**

Among boys in early adolescence, physical exercise habits were more persistent. Physical exercise promoted learning engagement, while higher learning engagement was linked to reduced internalizing symptoms. Among girls in early adolescence, internalizing symptoms showed greater persistence over time. Importantly, for both genders, internalizing symptoms undermine their learning engagement; physical exercise exerts a long-term protective effect against internalizing symptoms. Furthermore, for both genders, physical exercise enhanced learning engagement by mitigating internalizing symptoms.

**Supplementary Information:**

The online version contains supplementary material available at 10.1186/s13034-025-00965-7.

## Introduction

Learning constitutes the central task during adolescence. Learning engagement refers to the positive, fulfilling, and sustained psychological state students exhibit during the learning process [1]. It is widely recognized as a core dynamic assessment indicator of adolescents’ academic achievement potential and a crucial predictor of their mental health throughout their development [2]. Empirical evidence indicates that learning engagement is linked to positive emotional experiences [3], better academic performance [4], and lower levels of internalizing and externalizing symptoms among adolescents [5]. Conversely, a lack of engagement in learning activities is associated with adverse outcomes such as poor academic achievement [6], school disaffection [7]. Hence, investigating the factors influencing adolescents’ learning engagement holds significant importance for promoting their psychological well-being.

Adolescence represents a peak period for the emergence of internalizing symptoms [8]. Internalizing symptoms refer to the various emotional disturbances arising from maladaptation, primarily manifested as depression and anxiety [9]. Research indicates that a negative psychological state often impedes high learning engagement [10, 11]. Conversely, individuals exhibiting high learning engagement tend to experience fewer negative emotions [12]. Unlike the detrimental effects of internalizing symptoms, physical exercise serves as an effective intervention to promote learning engagement. Its characteristic cost-effectiveness and minimal adverse effects render it an increasingly favored tool for enhancing adolescent mental health [13]. Existing research demonstrates that ensuring adequate physical exercise among adolescents not only promotes their psychological well-being [13, 14] but is also crucial for the development of their learning abilities [15]. While existing research has yielded preliminary insights into the associations among physical exercise, internalizing symptoms, and learning engagement, most investigations have been restricted to pairwise relationships [13, 16–18]. A significant gap persists in examining the longitudinal relationships among all three variables and their potentially underlying mediating pathways. To address this gap, the present study adopts an exploratory research paradigm, utilizing a longitudinal design to investigate the longitudinal relationships among physical exercise, internalizing symptoms, and learning engagement, as well as potential mediating pathways among these three variables.

## Literature review

### Physical exercise and learning engagement

Physical exercise could be a contributing factor in enhancing learning engagement. According to embodied cognition theory, the process of cognitive involvement in learning occurs not solely within the brain but emerges from the dynamic interplay between the body, environment, and mind [19]. Physical exercise extracts individuals from static learning environments, shifts contexts through bodily movement, and restores depleted cognitive resources in adolescents during this process, thereby enhancing their learning engagement [20]. Cross-sectional evidence demonstrates the positive impact of physical exercise on learning engagement [16]. It is possible that physical exercise also has a long-term positive effect on learning engagement. According to the broaden-and-build theory, positive emotions can expand individuals’ thinking-action range, and accumulate lasting psychological resources through the “broaden-and-build” mechanism. Over time, these resources will be transferred to other fields [21]. Specifically, individuals who participate in long-term physical exercise could not only obtain positive emotional experiences, but also construct lasting personal physical, cognitive, psychological, and social resources through positive emotions. The accumulation of these resources benefits individual development, and its benefits also possibly extend to learning, work, and other aspects. The results of a meta-analysis that included 38 studies also indicate that physical exercise has a positive predictive effect on learning engagement [15].

The Integrative Model of Engagement likewise suggests a potential bidirectional relationship between physical exercise and learning engagement. The theory posits that learning engagement emerges from dynamic interaction between the environment and persons, influenced by both internal and external factors [12]. More specifically, the physical exercise process facilitates the gradual establishment and cultivation of certain individual resources, such as enhancing social [22], connections improving cognitive function, and strengthening self-control capacity [23] etc. These resources meet the internal needs of adolescents’ learning engagement development. Furthermore, the Integrative Model of Engagement further proposes resilience mechanisms within learning engagement, whereby students employ positive coping behaviors to mitigate daily stressors [12]. Specifically, it is possible that highly engaged students are more inclined to adopt physical exercise as a strategy to combat academic pressure. However, to our knowledge, very limited empirical evidence demonstrates either a positive effect of learning engagement on physical exercise or a bidirectional relationship between these constructs over time, and therefore, will be addressed in the present study.

### Physical exercise and internalizing symptoms

Physical exercise can be a protective factor against internalizing symptoms. According to emotion regulation theory, physical exercise not only provides a robust defense for mental health but also serves as an effective countermeasure against negative emotions [24]. Adolescents experiencing internalizing symptoms exhibit a propensity for self-doubt and self-blame; these negative cognitive patterns frequently exacerbate pre-existing symptoms. Physical exercise facilitates liberation from unfavorable environments and aids in redirecting attention toward positive stimuli [25]. Previous cross-sectional study have demonstrated that physical exercise can effectively alleviate internalizing symptoms [13]. Longitudinal studies also have demonstrated that long-term positive effect of physical exercise on internalizing symptoms [26–28].

On the other hand, internalizing symptoms contribute to reduced physical exercise participation among adolescents. Engaging in physical exercise represents an effortful adoption process lacking psychological shortcuts [29]. Sufficient motivation serves as an essential intrinsic driver for initiating this behavior—a drive frequently compromised among individuals experiencing internalizing symptoms [30]. Clinical research indicates that individuals with depression and anxiety frequently exhibit diminished behavioral motivation due to maladaptive cognitive patterns [30].

Theoretically, a bidirectional relationship appears to exist between physical exercise and internalizing symptoms over time. However, the directionality of the relationship between physical exercise and internalizing symptoms remains less clear. Previous studies using cross-lagged panel models (CLPM) have reported bidirectional [17] or non-significant [18] relationships between physical exercise and internalizing symptoms. Given that CLPM conflate between-person and within-person effects, potentially biasing results, recent studies have employed random-intercept cross-lagged panel models (RI-CLPM) to examine the relationship between physical exercise and internalizing symptoms. A longitudinal cohort study employing RI-CLPM revealed bidirectional relationships between physical exercise and depression [28]. In contrast, another RI-CLPM investigation found only unidirectional prediction from physical exercise to depression [26]. An additional RI-CLPM study demonstrated negative prediction from depression to physical exercise, while physical exercise failed to predict depressive symptoms [31]. Collectively, the relationship between physical exercise and internalizing symptoms remains incompletely understood.

### Internalizing symptoms and learning engagement

Internalizing symptoms contribute to diminished learning engagement among adolescents. The conservation of resources theory suggests that continued depletion of psychological resources will force adolescents to adopt resource defense strategies [32]. The development of internalizing symptoms accelerates the depletion of cognitive psychological resources in adolescents, which in turn makes it difficult for adolescents to stay vigorous during learning activities and to suppress their attention to distracting stimuli [33]. Previous studies have found that depression symptoms such as energy in efficiency and sleeping problems undermine individuals’ physiological well-being, and reduce their enthusiasm for learning [10]. Adolescents with anxiety symptoms are more likely to show boredom and low learning engagement in their studies [11].

Learning engagement serves as a protective factor against internalizing symptoms. The resilience mechanism within the Integrative Model of Learning Engagement proposes that students exhibiting higher learning engagement demonstrate a greater capacity to establish and maintain positive relationships [12]. These relationships, in turn, enhance psychological resilience and mitigate internalizing symptoms. The academic incompetence hypothesis also posits that deficient learning abilities constitute the primary cause of internalizing symptoms [34]. The long-term relationship between internalizing symptoms and learning engagement is likely bidirectional. One cross-lagged study found a bidirectional relationship between depression and learning burnout [35], while another study also revealed a bidirectional relationship between well-being and learning engagement [36]. Although such evidence demonstrates connections between emotions and learning, most findings regarding internalizing symptoms and learning engagement remain indirect. Therefore, this study will directly examine the longitudinal relationship between internalizing symptoms and learning engagement.

### Gender differences in physical exercise, internalizing symptoms, and learning engagement

It is possible that the relationship between physical exercise, internalizing symptoms, and learning engagement exhibits gender-specific variations. Gender role theory posits that gender roles influence individual behavior through gender identity, with distinct gender role expectations leading boys to demonstrate superior emotion regulation efficacy and heightened motivation for physical exercise compared to girls [37]. Evolutionary perspectives on sexual selection suggest adolescent girls exhibit greater sensitivity to stress and interpersonal relationships, alongside a heightened propensity for ruminating on negative experiences relative to boys [38]. This implies that girls presumably engage in less physical exercise, demonstrate elevated levels of internalizing symptoms, and encounter greater challenges in learning engagement due to sociocultural gender influences compared to boys. However, empirical findings present inconsistencies. Some studies indicate adolescent boys report higher levels of internalizing symptoms [39] and lower levels of learning engagement than girls [40], despite both genders sharing similar developmental trajectories in learning engagement [41]. Additional studies have examined gender differences in these relationships. For instance, One study found that physical exercise had a stronger negative association with internalizing symptoms in boys compared to girls [42]. Conversely, another study found a stronger negative association between physical exercise and internalizing symptoms among girls [43]. Although prior research has documented gender differences in these variables, investigations into gender effects on the relationships between internalizing symptoms and learning engagement, as well as between physical exercise and learning engagement, remain scarce. Thus, examining the role of gender differences in the longitudinal relationships among physical exercise, internalizing symptoms, and learning engagement will help address this critical gap.

### Current study

**Fig. 1:**
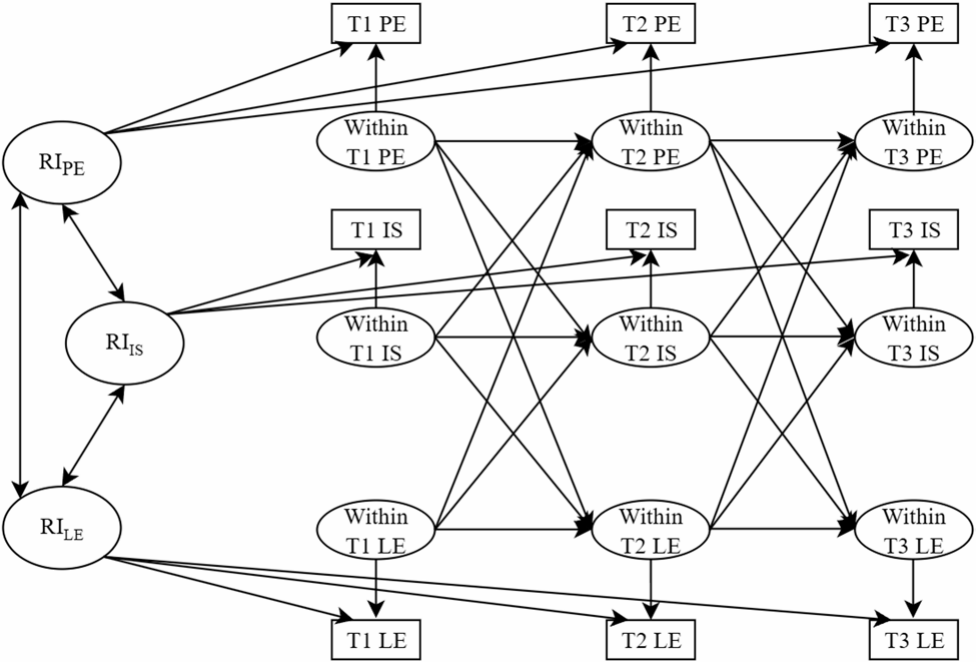
Hypothetical Model of Physical Exercise, Internalizing Symptoms, and Learning Engagement. *Note*: RI represents random intercept; PE represents physical exercise; IS represents internalizing symptoms; LE represents learning engagement

Although previous research has preliminarily established the associations among physical exercise, internalizing symptoms, and learning engagement, several critical issues warrant further investigation. First, although existing research has examined pairwise longitudinal associations among physical exercise, internalizing symptoms, and learning engagement, integrated investigations of the longitudinal relationships among all three variables remain scarce. Research is particularly lacking on potential longitudinal mediating mechanisms operating among these three variables. Second, although most investigations have employed CLPM to examine variable relationships, CLPM findings face increasing scrutiny due to conflating between-person and within-person effects. Compared to CLPM, the RI-CLPM distinguishes stable individual differences from dynamic intraindividual fluctuations, significantly improving the accuracy of longitudinal relationship estimation [44]. Finally, as indicated above, it is possible that significant gender differences both within and between the variables of physical exercise, internalizing symptoms, and learning engagement. However, However, gender is mostly a control variable in previous research [13, 16, 26], which may introduce bias into the findings. Conceptualizing gender as a grouping variable for comparative analysis can reveal differential relational patterns across gender groups, yielding more robust and gender-specific conclusions. Therefore, the primary objectives of this study are: (1) To examine within-person dynamics and underlying mediating mechanisms among physical exercise, internalizing symptoms, and learning engagement; (2) To investigate gender differences in their longitudinal relationships through multi-group comparisons, identifying specific paths exhibiting gender heterogeneity. (The hypothetical model is shown in Fig. 1)

## Methods

### Participants

Using a cluster convenience sampling method, we selected seventh and eighth grade students from three middle schools in Henan Province, China, and conducted three follow-up surveys at one-year intervals in December 2023 (T1), June 2024 (T2), and December 2024 (T3). At the first time point (T1), 1,789 questionnaires were distributed, and 1,601adolescents participated in the study, with an effective response rate of 94. 46%. The losses occurred due to reasons such as school transfers and illness, resulting in the absence of 45 students in the second survey and 34 in the third. After excluding 11 participants due to missing or repeated responses, the final sample comprised 1,601 students for analysis. Using the R package *simsem* for Monte Carlo simulation [45], under the conditions of within-person effects of 0.1–0.2 and an ICC of 0.50, 424 participants are required to achieve a statistical power of 0.80 (α = 0.05), which indicates that the sample size of this study met the statistical requirements. In this study, the participants ranged in age from12 to 16 years old, with an average age of 13. 92 and a standard deviation of 0. 72. Among them, 870 were boys and 731 were girls.

### Measurement tools

#### Physical exercise

The physical exercise rating scale was used to as an indicator of adolescent physical exercise [46], consisting of three evaluation indicators: exercise duration, exercise intensity, and exercise frequency. For example, a question describing the intensity of exercise is “What sports activities have you frequently participated in recently?” Each indicator is rated on a 5-level scale, and the physical exercise amount is calculated by the formula of “physical exercise intensity × (physical exercise duration-1) × physical exercise frequency”. The scale yields a maximum score of 100 points. Participants are classified into three physical exercise levels based on established cutoffs: low (≤ 19 points), moderate (20–42 points), and high (≥ 43 points). Previous studies have shown that this scale performs well in measuring the physical exercise level of Chinese samples [16]. According to Liang’s initial research paradigm [46], this study conducted a reliability retest. At each measurement wave (T1-T3), three randomly selected student groups underwent retesting after a one-week interval. The test-retest reliability coefficients were 0.757 (T1), 0.739 (T2), and 0.713 (T3), This demonstrates that the reliability of the questionnaire is acceptable.

### Internalizing symptoms

The Chinese version of the Depression-Anxiety-Stress scale − 21, originally designed by Lovibond et al. [47], was adopted to evaluate the depression, anxiety, and stress status of the participants. The scale comprises 21 items presented as statements describing individuals’ recent negative emotional experiences or corresponding physiological responses, such as “I felt depressed and gloomy.” It’s Depression and Anxiety-Stress sub-scales contain seven items each, with a four-grade scale from 0 to 3. Higher scores indicate more severe depressive and anxiety symptoms, as well as greater perceived stress. Previous studies have shown that this scale performs well in measuring internalizing symptoms in Chinese adolescent population [48]. The construct validity of internalizing symptoms was favorable at the three time points of this study (T1: RMSEA = 0. 079, CFI = 0. 917, TLI = 0. 905, SRMR = 0. 038; T2: RMSEA = 0. 084, CFI = 0. 916, TLI = 0. 905, SRMR = 0. 038; T3: RMSEA = 0. 079, CFI = 0. 939, TLI = 0. 931, SRMR = 0. 029). The Cronbach’s α coefficients for the three time points of internalizing symptoms are 0. 956, 0. 962, and 0. 972, respectively.

### Learning engagement

The Utrecht Work Engagement Scale, originally designed by Schaufeli et al. [1]. and revised by Fang Latina et al. [49], was employed to access leaning engagement. It consists of 17 questions across three dimensions: vigor, dedication, and absorption. A sample item reads: “I feel energetic while studying.” The 7-point Likert scale was adopted, with higher scores indicating higher level of adolescents’ learning engagement. This scale has shown good performance in measuring learning engagement in Chinese samples [16]. In this study, the construct validity of learning engagement was favorable at the three time points (T1: RMSEA = 0. 095, CFI = 0. 936, TLI = 0. 924, SRMR = 0. 037; T2: RMSEA = 0. 100, CFI = 0. 943, TLI = 0. 933, SRMR = 0. 032; T3: RMSEA = 0. 091, CFI = 0. 960, TLI = 0. 953, SRMR = 0. 020). The Cronbach’s α coefficients for the three time points of learning engagement are 0. 966, 0. 975, and 0. 983, respectively.

### Data analysis method

The study analyzed the data with SPSS 27. 0 and Mplus 8. 10. First, descriptive statistics, correlation analysis, and common method bias assessment were conducted on each subject variable with SPSS 27. 0. Second, measurement invariance testing was conducted on the three-wave measurement data using Mplus 8. 10. Subsequently, based on preliminary correlation analyses and established measurement invariance, we constructed both CLPM and RI-CLPM using Mplus 8. 10, then systematically compared their model fit indices. Finally, a multi-group structural equation modeling approach was adopted to examine potential gender differences in the relationships between physical exercise, internalizing symptoms, and learning engagement [50]. Following the recommendations of Mulder and Hamaker [50], two nested models were constructed: Model 1 (M1) freely estimated all path coefficients across groups, while Model 2 (M2) constrained the autoregressive and cross-lagged path coefficients to be equal between groups. If M1 demonstrated significantly better fit indices than M2, this would indicate that the path coefficients could not be constrained to equality across groups, suggesting gender differences in these relationships. Conversely, if no significant improvement in model fit was observed, it would imply no substantial differences between groups.

## Results

### Descriptive statistics and correlation analysis

Table 1 presents the descriptive analysis and correlation matrix for physical exercise, internalizing symptoms, and learning engagement. The results indicate: At different time points, the same variables consistently showed significant positive correlations (Physical exercise: *r* = 0.40–0.57, *p* < 0.001; Internalizing symptoms: *r* = 0.61–0.74, *p* < 0.001; Learning engagement: *r* = 0.46–0.66, *p* < 0.001). Across all three measurements, physical exercise and internalizing symptoms were significantly negatively correlated (*r* = -0.10 to -0.27, *p* < 0.001), physical exercise and learning engagement showed significant positive correlations (*r* = 0.06 to 0.20, *p* < 0.01), and internalizing symptoms and learning engagement were significantly negatively correlated (*r* = -0.33 to -0.49, *p* < 0.01). This meets the prerequisite hypothesis of cross-lagged analysis.

**Table 1:** Descriptive statistics and correlation analysis

Variable	M ± SD	1	2	3	4	5	6	7	8	9
1. TI PE	21.57 ± 21.19	1								
2. T2 PE	24.06 ± 20.97	0.57^***^	1							
3. T3 PE	23.71 ± 20.17	0.40^***^	0.46^***^	1						
4. T1 IS	0.84 ± 0.66	-0.27^***^	-0.19^**^	-0.13^***^	1					
5. T2 IS	0.82 ± 0.65	-0.27^***^	-0.22^***^	-0.14^***^	0.68^***^	1				
6. T3 IS	0.73 ± 0.57	-0.22^***^	-0.19^***^	-0.10^***^	0.61^***^	0.74^***^	1			
7. T1 LE	4.13 ± 1.35	0.18^***^	0.13^***^	0.06^**^	-0.42^***^	-0.37^***^	-0.33^***^	1		
8. T2 LE	4.23 ± 1.41	0.20^***^	0.19^***^	0.10^***^	-0.42^***^	-0.49^***^	-0.42^**^	0.66^***^	1	
9. T3 LE	4.05 ± 1.51	0.16^***^	0.16^***^	0.14^***^	-0.34^***^	-0.42^***^	-0.40^***^	0.46^***^	0.55^***^	1

PE represents physical exercise; IS represents internalizing symptoms; LE represents learning engagement ;*p < 0.05, **p < 0.01, ***p < 0.001

### Measurement invariance test

This study sequentially constructed four nested models for longitudinal measurement invariance testing: a configural invariance model, a weak invariance model (factor loading invariance), a strong invariance model (item intercept invariance), and a strict invariance model (error variance invariance) [52]. Measurement invariance was established if changes in CFI and RMSEA were below the threshold of |Δ| < 0.01 [53]. When discrepancies between fit index changes occurred, precedence was given to the RMSEA change. The measurement invariance test results are presented in Table 2, which also reports chi-square test results. Given that chi-square tests are overly sensitive to sample size, these results should be interpreted as supplementary reference rather than primary evidence.

As shown in Table 2, both internalizing symptoms and learning engagement demonstrated strict invariance. This indicates stable factor loadings, item means, and error variances for the constructs of internalizing problems and learning engagement over time. Physical exercise met the criteria for weak invariance, confirming consistent participant understanding of the physical exercise construct across measurement waves. However, strong invariance was not achieved, suggesting that interpretation of specific items changed over time, resulting in shifts in item means. Although the physical exercise scale is a non-constructed scale, longitudinal studies have validated its strong measurement invariance [54]. In the current study, the failure to establish strong invariance for this scale may be developmentally attributed to distinct stages. During middle school, adolescents’ exercise intensity, duration, and frequency are susceptible to external influences [55]. At the university stage, however, these parameters become more self-regulated and remain relatively stable [54].

**Table 2:** Longitudinal measurement invariance test for physical exercise, internalizing symptoms, and learning engagement

Variable	Model	χ^2^	df	CFI	TLI	RMSEA	SRMR	Model compare	Δχ^2^	Δdf	*p*	ΔCFI	ΔRMSEA
PE													
	M1: Configural invariance	59.598	15	0.987	0.969	0.043	0.023						
	M2: Weak invariance	81.170	19	0.982	0.966	0.045	0.032	M2-M1	21.572	4	< 0.05	-0.003	0.002
	M3: Strong invariance	168.921	23	0.958	0.934	0.063	0.040	M3-M2	87.751	4	< 0.05	-0.024	0.018
IS													
	M1: Configural invariance	9696.018	1815	0.909	0.902	0.052	0.033						
	M2: Weak invariance	9992.590	1855	0.906	0.900	0.052	0.036	M2-M1	296.572	40	< 0.05	-0.003	0.000
	M3: Strong invariance	10365.607	1895	0.902	0.899	0.053	0.037	M3-M2	374.381	40	< 0.05	-0.004	0.001
	M4: Strict invariance	10963.688	1937	0.895	0.894	0.054	0.047	M4-M3	598.081	42	< 0.05	-0.008	0.001
LE													
	M1: Configural invariance	8310.478	1161	0.930	0.923	0.062	0.036						
	M2: Weak invariance	8372.049	1193	0.930	0.925	0.061	0.036	M2-M1	61.571	32	< 0.05	0.000	-0.001
	M3: Strong invariance	8783.900	1225	0.926	0.923	0.062	0.037	M3-M2	411.851	32	< 0.05	-0.004	0.001
	M4: Strict invariance	10317.627	1259	0.911	0.910	0.067	0.044	M4-M3	1533.727	34	< 0.05	-0.015	0.005

PE represents physical exercise; IS represents internalizing symptoms; LE represents learning engagement

### RI-CLPM analysis of physical exercise, internalizing symptoms, and learning engagement

**Fig. 2:**
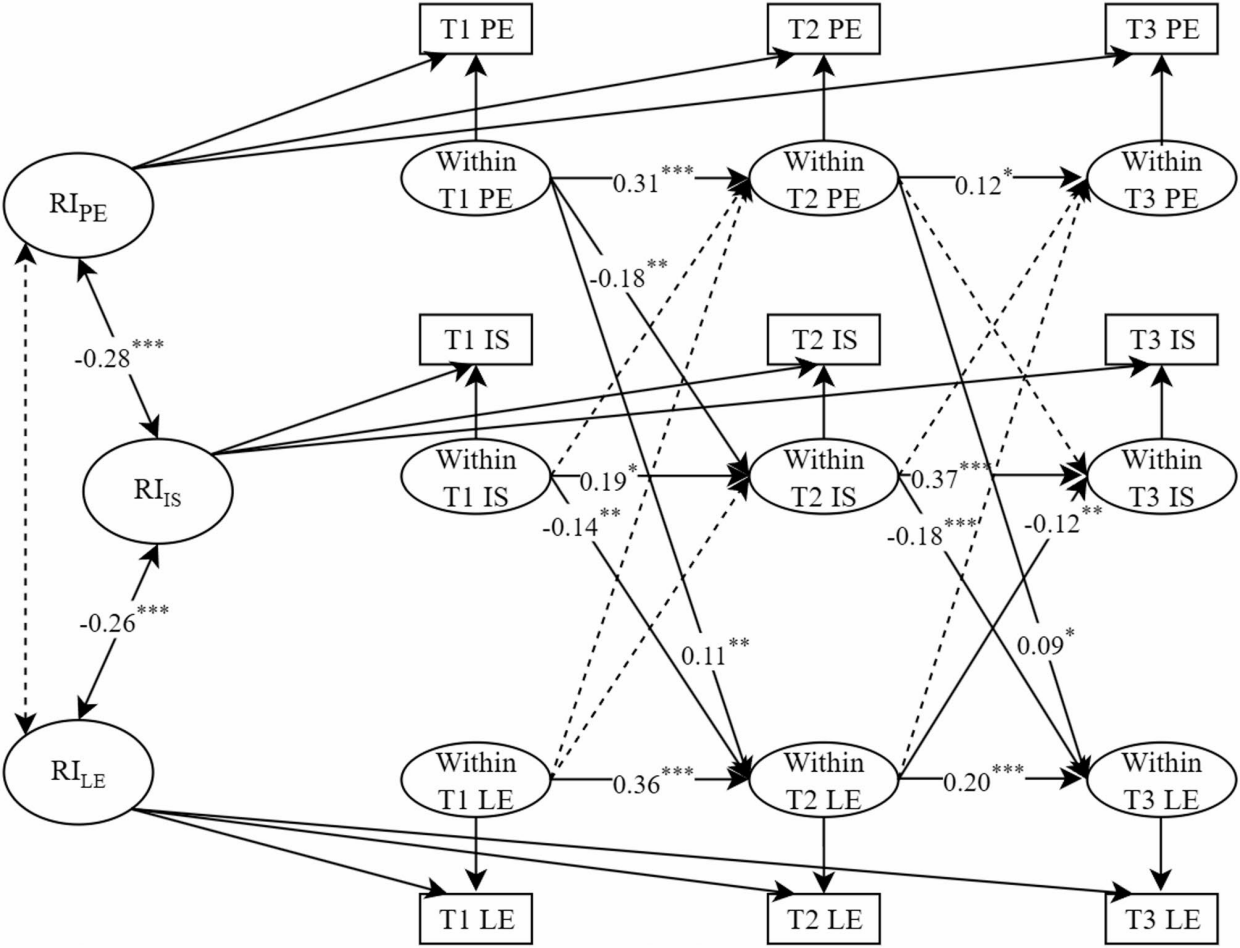
RI-CLPM Results of Physical Exercise, Internalizing Symptoms, and Learning Engagement. *Note*: The coefficients in the figure are standardized pathway coefficients; RI represents random intercept; PE represents physical exercise; IS represents internalizing symptoms; LE represents learning engagement; *p < 0.05, **p < 0.01, ***p < 0.001

The RI-CLPM demonstrated excellent fit to the data: *χ*^*2*^ = 6. 351, *df* = 3, *χ*^*2*^*/df* = 1. 374, RMSEA = 0. 026, CFI = 0. 999, TLI = 0. 993, SRMR = 0. 008. To determine whether the data were more suitable for the RI-CLPM, we additionally constructed a CLPM (See Table 1 of the Supplementary Materials). The model fit statistics are as follows: *χ*^*2*^ = 165.298, *df* = 9, *χ*^*2*^*/df* = 18.366, RMSEA = 0. 104, CFI = 0. 971, TLI = 0. 893, SRMR = 0. 026. This clearly demonstrates the superiority of the RI-CLPM over the CLPM in terms of model fit (ΔCFI = 0.106, ΔRMSEA = 0.078). Using comparative model methods, the analysis demonstrated that the RI-CLPM exhibited significantly superior model fit indices compared to the CLPM (Δ*χ*^*2*^ = 158.947, Δ*df* = 6, *P*<0.001). According to Chen’s criterion [52], this study’s data demonstrate substantially superior fit with the RI-CLPM framework. Therefore, this study employs the RI-CLPM to explore the longitudinal relationships among physical exercise, internalizing symptoms, and learning engagement. Figure 2 shows the RI-CLPM results of the longitudinal relationships among physical exercise, internalizing symptoms, and learning engagement. The results from the RI-CLPM revealed that: (1) at the between-person level, there were significant negative correlation between physical exercise and internalizing symptoms; (2) there were significant negative correlation between internalizing symptoms and learning engagement; (3) the correlation between physical exercise and learning engagement is not significant. At the within-person level, physical exercise, the internalizing symptoms, and learning engagement each demonstrate cross-temporal stability (All cross-lagged paths are shown in Table 3; mediating effect see Table 4).

**Table 3:** Standardized within-person concurrent associations coefficients for RI-CLPM among physical exercise, internalizing symptoms and learning engagement

Pathway	T1→T2			T2→T3		
	β	SE	*p*	β	SE	*p*
PE→PE	0.31	0.05	< 0.001	0.12	0.06	< 0.05
IS→IS	0.19	0.08	< 0.05	0.37	0.06	< 0.001
LE→LE	0.36	0.05	< 0.01	0.20	0.05	< 0.001
PE→IS	-0.18	0.06	< 0.01	-0.01	0.03	0.739
PE→LE	0.36	0.05	< 0.001	0.09	0.04	< 0.05
IS→LE	-0.14	0.04	< 0.01	-0.18	0.04	< 0.001
LE→IS	-0.07	0.06	0.209	-0.12	0.04	< 0.01
IS→PE	-0.02	0.04	0.622	0.03	0.05	0.532
LE→PE	0.07	0.04	0.144	0.08	0.05	0.133

**Table 4:** Longitudinal mediation analysis

Pathway	Effect	SE	*p*	95% CI	
				Boot LLCI	Boot ULCI
T1 PE → T2 IS → T3 LE	0.03	0.01	< 0.01	0.007	0.061
T1 PE → T2 LE → T3 IS	-0.01	0.008	0.06	-0.03	0.001

### Gender differences in physical exercise, internalizing symptoms, and learning engagement

The multi-group structural equation modeling was applied to examine potential gender differences in the longitudinal relationships among physical exercise, internalizing symptoms, and learning engagement in early adolescence [50]. First, we specified a constrained model in which all autoregressive and cross-lagged path coefficients were set equal across gender groups. Second, an unconstrained model was established allowing separate estimation of autoregressive and cross-lagged paths for boys and girls. Model fit was then compared using chi-square difference statistics to determine significant gender differences. Where significant effects emerged, Wald tests were conducted to identify specific paths with statistically significant differences between genders. If significant differences were found, Wald tests were conducted to identify which specific paths differed significantly between boys and girls. The results showed that the constrained model fitted well (*χ*^*2*^*/df* = 2.814, RMSEA = 0.048, CFI = 0.992, TLI = 0.976, SRMR = 0.023), with significant differences (Δ*χ*^*2*^ = 61.711, Δ*df* = 18, *P*<0.001) to the unconstrained model (*χ*^*2*^*/df* = 0.973, RMSEA = 0.000, CFI = 1.000, TLI = 1.000, SRMR = 0.008). This indicates significant gender differences in physical exercise, internalizing symptoms, and learning engagement. Therefore, this study conducted further examinations of the pathways exhibiting significant gender differences (The gender difference test in the path is shown in the Table 5).

**Table 5:** Model test of gender differences across pathways

Pathway	Wald’s χ^2^	*p*	Boys		Girls	
			β	*p*	β	*p*
T1 PE → T2 PE	10.464	< 0.001	0.37	< 0.001	0.06	0.396
T1 PE → T2 LE	4.842	< 0.05	0.17	< 0.01	-0.02	0.669
T2 IS → T3 IS	14.642	< 0.001	0.11	0.238	0.51	< 0.001
T2 LE → T3 IS	4.599	< 0.05	-0.25	< 0.01	-0.03	0.495
T2 PE → T3 LE	7.530	< 0.001	0.18	< 0.001	-0.04	0.396

## Discussion

This study employed RI-CLPM to examine within-person relationships and potential mediating pathways among physical exercise, internalizing symptoms, and learning engagement. This study extends previous research and offers methodological innovation through rigorous separation of within-person processes from stable individual differences. Multi-group comparisons further revealed gender-specific patterns in these dynamics, providing a robust empirical basis for developing targeted interventions. The results indicated that: At the within-person level, physical exercise, internalizing symptoms, and learning engagement all demonstrated significant within-person stability. Physical exercise negatively predicted subsequent internalizing symptoms. Physical exercise positively predicted subsequent learning engagement. Internalizing symptoms and learning engagement exhibited reciprocal negative effects on each other over time. Internalizing symptoms longitudinally mediated the relationship between physical exercise and learning engagement. Multi-group comparisons by gender revealed significant variations in the longitudinal relationships among physical exercise, internalizing symptoms, and learning engagement.

### The long-term positive association between physical exercise and learning engagement

This study found that within-person increases in physical exercise were associated with later increases in learning engagement. These findings indicate that physical exercise has both short-term and long-term beneficial effects on adolescents’ learning engagement, consistent with the broaden-and-build theory of positive emotions [21]. Regular physical exercise fosters enduring positive emotion that will continuously expand individuals’ physical, psychological, and cognitive resources [14]. The development of physical resources grants adolescents vitality and sustained mental engagement in the learning process [56]. Moreover, physical exercise activates the function of the prefrontal cortex [57]. The activation of prefrontal cortical functions is critical for focused learning engagement [58]. Finally, the expansion of psychological resources is also a key mechanism through which physical exercise enhances learning engagement. Physical exercise boosts the development of many positive psychological assets, including psychological resilience [59], self-efficacy [60] etc., which have been identified as critical predictors of learning engagement [12]. However, this study did not detect a bidirectional relationship between physical exercise and learning engagement. This finding presumable stems from Chinese exam-oriented education system, where adolescents face overwhelming academic demands. Students with high learning engagement likely have heavy learning workloads, leaving little time for physical exercise [61].

### The long-term negative association between physical exercise and internalizing symptoms

This study found that baseline levels of physical exercise significantly and negatively predicted subsequent internalizing symptoms, these findings align with previous research [26]. First, physical exercise enhances adolescents’ emotion regulation capabilities [62]. This enables them to more effectively cope with and manage emotions when facing stress and adversity, thereby reducing the occurrence of internalizing symptoms such as anxiety and depression [13, 25]. Furthermore, adolescents who engage in physical exercise often demonstrate stronger social adaptation and more positive interpersonal relationship-building skills in social contexts [22]. This reduces the negative psychological impact associated with feelings of isolation and social exclusion. Finally, physical exercise can alleviate internalizing symptoms by regulating the neuroendocrine system and promoting brain development [63]. Notably, internalizing symptoms did not diminish physical exercise levels, a finding largely consistent with the existing literature [26]. This finding revealed that, within Chinese collectivist cultural framework emphasizing harmony, overt expression of internalizing symptoms is often perceived as “face-threatening” behavior that risks social harmony or imposes familial burdens [64]. Under this model, the internalizing symptoms may not directly reduce participation in physical exercise.

This study also revealed a non-significant predictive effect of physical exercise at T2 on internalizing symptoms at T3. The Process-Person-Context-Time (PPCT) model of the Bioecological Theory posits that an individual’s inherent characteristics, interactions with environments (such as family and school), and the timing and frequency of these interactions collectively form an interdependent system that shapes development [65]. In other words, internalizing symptoms are influenced by distinct factors across different developmental stages [66]. This dynamic introduces the possibility of interference from other variables when examining how physical exercise affects internalizing symptoms [31]. Nevertheless, the protective role of physical exercise against internalizing symptoms is well-established, and substantial evidence underpins this study [13, 26]. Future research should employ longitudinal designs with extended timeframes and higher-frequency assessments to further investigate this protective effect.

### The bidirectional associations between internalizing symptoms and learning engagement

This study also revealed bidirectional negative predictions between internalizing symptoms and learning engagement. First, the internalizing symptoms at earlier time points negatively predicted the reduced subsequent learning engagement. This negative association presumably stems from the persistent depletion of emotional resources caused by internalizing symptoms, which weakens individuals’ self-monitoring capacity, undermines attentional stability, and exhausts volitional control resources [32]. Such self-regulation failures directly impair the maintenance of deep learning states. This finding aligns with Schaufeli’s original conceptualization of learning engagement as a positive psychological state that significantly promotes students’ learning engagement; on the contrary, a negative psychological state triggers learning fatigue [1]. Second, the higher initial learning engagement negatively predicted the subsequent internalizing symptoms. The academic incompetence model posits that unsuccessful learning experiences increase vulnerability to internalizing symptoms [34]. Notably, learning engagement not only significantly predicts the learning achievement of adolescents, but also mitigates their negative learning experiences and enhances their sense of happiness [36]. Compared to adolescents with lower learning engagement, adolescents with higher learning engagement often suffer fewer internalizing symptoms and academic failures [41].

This study also revealed a non-significant predictive effect of learning engagement at T1 on internalizing symptoms at T2. This finding indicates that during early adolescence, the protective effect of learning engagement against internalizing symptoms is less pronounced than the risk that internalizing symptoms, once increased, will subsequently lead to a decline in learning engagement. According to the integrated developmental model of learning engagement, internalizing symptoms represent a distal outcome of learning engagement [12]. The model posits that the influence of learning engagement on internalizing symptoms only gradually manifests over time.

### The longitudinal mediating role of internalizing symptoms

This study examined the potential mechanisms underlying the longitudinal relationship between physical exercise, internalizing symptoms, and learning engagement. It found that internalizing symptoms serve as a mediating factor between physical exercise and learning engagement. This result supports the hierarchy of needs theory [67], positing that physical exercise—as a physiological necessity—addresses health needs to reduce internalizing symptoms, thereby facilitating self-actualization through enhanced learning engagement. Previous research has confirmed that physical exercise can reduce internalizing symptoms through physiological and psychological pathways [62, 68], thereby promoting self-actualization needs.

### Gender differences in longitudinal relationships among physical exercise, internalizing symptoms, and learning engagement

To enhance the robustness of the findings, multi-group comparisons were employed to examine longitudinal relationships between physical exercise, internalizing symptoms, and learning engagement.

Compared to girls, boys demonstrated greater cross-temporal stability in physical exercise levels (as measured by autoregressive coefficients). In traditional Chinese culture, boys are more likely to gain pleasure from physical exercise, use it for self-expression, prove themselves, and enjoy competing with others. While girls tend to view exercise instrumentally due to traditional Chinese gender norms, which undermines their capacity motivation [69].

Physical exercise exerted significant longitudinal positive effects on learning engagement exclusively among boys, whereas no significant effects were observed in girls. Gender role theory posits that the gender-role expectations formed during gender socialization serves as a moderating factor [37]. Specifically, Boys are often encouraged to release academic pressure through physical exercise, while girls may choose non-physical emotion regulation. This differentiated selection of coping strategies further reinforces the development of gender-specific pathways.

Girls demonstrated significantly greater longitudinal stability in internalizing symptom levels compared to Boys. This result aligns with the evolutionary sexual selection theory: adolescent girls experience earlier physiological maturation than their male peers. Such developmental difference between physical and psychological growth often lead to girls’ stronger emotional perception, enhanced perceptual sensitiveness to external stimuli such as stressors and interpersonal interactions, and greater tendency to meta-cognitive reflection on negative experiences [38]. The interplay between their complex and ever-changing affective system and delicate and sensitive perceptual traits leads to the persistent development of internalizing symptoms among girls [70].

Boys exhibited stronger cross-temporal stability in physical exercise, while girls showed greater stability in internalizing symptoms. The association between physical exercise and learning engagement was significant only in boys. This may be because in girls’ early adolescence, learning engagement shows weaker effect on their internalizing symptoms, a developmental period when emotional well-being is often closely related to relational networks [71]. When learning engagement fails to effectively integrate with the peer support system, its protective function against internalizing symptoms may be partially eliminated by interpersonal sensitivity.

### Limitations and significance

This study has several limitations that should be acknowledged. First, physical exercise was measured through questionnaires rather than behavioral indicators; both internalizing symptoms and school engagement were measured by self-rated scales without other-rated indicators. The data sources and measurement indicators were both limited in diversity. Future research should adopt more objective and diversified measurement methods to examine the relationships among the three. Second, this study examined the impact of gender on physical exercise, internalizing symptoms, and learning engagement through multiple group comparisons, without including additional demographic variables, which may introduce slight biases. Future studies should consider factors like subjective socioeconomic status and school type that influence physical exercise, internalizing symptoms, and learning engagement. Finally, Given the high comorbidity between internalizing and externalizing symptoms [66], our study’s omission of externalizing symptoms as a covariate may introduce subtle biases. Future research examining the longitudinal relationships among physical exercise, internalizing symptoms, and learning engagement should incorporate externalizing symptoms to account for their potential confounding effects.

The theoretical significance of this study is reflected in two aspects. This study is the first to adopt an integrated perspective in examining the within-person relationships among physical exercise, internalizing symptoms, and learning engagement in adolescents, as well as their underlying mediating mechanisms. It thereby establishes a robust empirical foundation for future research. Second, this study disentangled the influence of gender on the longitudinal relationships and potential mediating mechanisms linking physical exercise, internalizing symptoms, and learning engagement, thereby providing empirical support for the nature of these longitudinal associations within different gender groups.

This study carries significant implications for preventive interventions targeting adolescent mental health. Middle schools should prioritize physical education by actively cultivating sustainable exercise habits to promote psychological well-being. Furthermore, schools must account for gender-specific patterns in physical activity outcomes. Rather than adopting uniform approaches, they should develop tailored strategies aligned with documented gender differences. Finally, we propose implementing structured activity breaks—specifically, brief physical exercise sessions after every four hours of academic work—as a potential strategy to enhance sustained learning engagement [72] and mitigate the accumulation of internalizing symptoms [73].

## Conclusion

Among boys in early adolescence, physical exercise habits were more persistent. Physical exercise promoted learning engagement, while higher learning engagement was linked to reduced internalizing symptoms. Among girls in early adolescence, internalizing symptoms showed greater persistence over time. Importantly, for both genders, internalizing symptoms undermine their learning engagement; physical exercise exerts a long-term protective effect against internalizing symptoms. Furthermore, for both genders, physical exercise enhanced learning engagement by mitigating internalizing symptoms.

## Supplementary Information

Below is the link to the electronic supplementary material.

Supplementary Material 1

## Data Availability

The data used in this study have been anonymized and securely stored. The research team will provide data for academic research upon reasonable request, which can be made by contacting the first author, Bohang Wei, at [593894259@qq. com](mailto:593894259@qq.com) .
