# Supplementary material for: Longitudinal relationships among early adolescent physical exercise, internalizing symptoms, and learning engagement: exploring within-person dynamics and the role of gender differences
Source: Child Adolesc Psychiatry Ment Health. 2025 Sep 29;19:104. doi: 10.1186/s13034-025-00965-7 (PMC12482010; doi:10.1186/s13034-025-00965-7)
Supplement: Supplementary file 1 — Supplementary Material 1 [file 13034_2025_965_MOESM1_ESM.docx]

**Supplementary Material**

**CLPM analysis of physical exercise, internalizing symptoms, and learning engagement**

The CLPM demonstrated excellent fit to the data: *χ^2^* = 165.298, *df* = 9, *χ^2^/df* = 18.366, RMSEA = 0. 104, CFI = 0. 971, TLI = 0. 893, SRMR = 0. 026. The results from the CLPM revealed that: (1) Physical exercise has a positive predictive effect on learning engagement; (2) physical exercise has a negative predictive effect on internalizing problems; (2) there is a bidirectional relationship between internalizing problems and learning engagement; (4) internalization plays a longitudinal mediating role between physical activity and learning engagement. Table 1 shows the CLPM results of the longitudinal relationships among physical exercise, internalizing symptoms, and learning engagement.

Table 1 Standardized concurrent associations coefficients for CLPM among physical exercise, internalizing symptoms and learning engagement.

| Pathway | T1→T2 | | | T2→T3 | | |
| --- | --- | --- | --- | --- | --- | --- |
|  | *β* | SE | *P* | *β* | SE | P |
| PE→PE | 0.56 | 0.024 | <0.001 | 0.45 | 0.025 | <0.001 |
| IS→IS | 0.61 | 0.023 | <0.001 | 0.70 | 0.020 | <0.001 |
| LE→LE | 0.58 | 0.024 | <0.001 | 0.45 | 0.025 | <0.001 |
| PE→IS | -0.08 | 0.021 | <0.001 | -0.02 | 0.018 | 0.260 |
| PE→LE | 0.05 | 0.021 | <0.01 | 0.03 | 0.021 | 0.076 |
| IS→LE | -0.16 | 0.023 | <0.001 | -0.18 | 0.024 | <0.001 |
| LE→IS | -0.09 | 0.023 | <0.001 | -0.06 | 0.022 | <0.01 |
| IS→PE | -0.03 | 0.024 | 0.170 | -0.03 | 0.027 | 0.170 |
| LE→PE | 0.01 | 0.026 | 0.531 | 0.003 | 0.028 | 0.908 |

Note: The coefficients in the figure are standardized pathway coefficients; RI represents random intercept; PA represents physical exercise; IS represents internalizing symptoms; LE represents learning engagement;
